# One-Year Clinical Outcome in Middle Eastern Patients with Atrial Fibrillation: The Jordan Atrial Fibrillation (JoFib) Study

**DOI:** 10.1155/2022/4240999

**Published:** 2022-04-13

**Authors:** Ayman Hammoudeh, Yousef Khader, Ramzi Tabbalat, Yahya Badaineh, Nazih Kadri, Haneen Shawer, Eyas Al-Mousa, Rasheed Ibdah, Batool A. Shawer, Imad A. Alhaddad

**Affiliations:** ^1^Department of Cardiology, Istishari Hospital, 44 Kindi Street, Amman 11954, Jordan; ^2^Department of Public Health, Jordan University of Science and Technology School of Medicine, 3030 Ramtha Street., Irbid 22110, Jordan; ^3^Cardiology Department, Abdali Hospital, 1 Al-Istethmar Street-Abdali Boulevard, Amman 11190, Jordan; ^4^Department of Internal Medicine, Cardiology Department, King Abdullah University Hospital, Irbid, Jordan; ^5^Department of Cardiology, Jordan Hospital, Queen Rania Street, Amman, Jordan

## Abstract

**Background:**

Prevention of stroke and systemic embolism (SE) prevention in patients with atrial fibrillation (AF) has radically changed in recent years. Data on contemporary utilization of oral anticoagulants (OACs) and cardiovascular outcome in Middle Eastern patients with AF are needed.

**Methods:**

The Jordan atrial fibrillation (JoFib) study enrolled consecutive patients with AF in Jordan from May 2019 through October 2020 and were followed up for one year after enrollment.

**Results:**

Overall, 2020 patients were enrolled. The mean age was 67.9 + 13.0 years. Nonvalvular (NVAF) was diagnosed in 1849 (91.5%) patients. OACs were used in 85.7% of high-risk patients with NVAF (CHA2DS2-VASc score>3 in women, and>2 in men), including direct OACs (DOACs) in 64.1% and vitamin K antagonists (VKA) in 35.9%. Adherence rate to the use of the same OAC agent was 90.6% of patients. One-year cardiovascular (CV) mortality was 7.8%, stroke/SE was 4.5%, and major bleeding events were 2.6%. Independent predictors for all-cause mortality in patients with NVAF were age>75 years, heart failure, major bleeding event, type 2 diabetes mellitus, study enrollment as an in-patient, and coronary heart disease. The use of OACs was associated with lower all-cause mortality. The strongest independent predictors for stroke/SE were high-risk CHA2DS2-VASc score and prior history of stroke.

**Conclusions:**

This study of Middle Eastern AF patients has reported high adherence to OACs. The use of OACs was associated with a lower risk for all-cause mortality. One-year rates of stroke and major bleeding events were comparable to those reported from other regions in the world.

## 1. Introduction

Atrial fibrillation (AF), the most common chronic cardiac arrhythmia in adults, affects 1-2% of the general population, accounts for one-third of hospitalizations for dysrhythmias, and is associated with high risk of significant long-term devastating sequelae of stroke, systemic embolism (SE), and mortality [1, 2]. There is evidence of progressive increases in overall burden of nonvalvular AF (NVAF) over the past few decades, with significant public health implications [3, 4]. Potential explanations for these secular trends include demographic transition to an inverted age pyramid with an increase in the proportions of older age groups, increase in age-related comorbidities and risk factors that contribute to the development of AF, and rise of cardiovascular disease burden in developing countries [3–5]. Moreover, the rate of utilization of clinical practice guidelines-recommended oral anticoagulant agents (OACs), direct OACs (DOACs) in particular, varies significantly between various regions in the world due to limited availability and high cost, despite their positive role that has led to a major change in the landscape of stroke and SE prevention in patients with AF [6, 7].

The great majority of the clinical studies and registries that evaluated the incidence of adverse events in patients with AF originated from Western and East Asian populations [8]. Furthermore, phase III studies that evaluated the efficacy of the DOACs in stroke prevention in patients with NVAF may not necessarily reflect the daily practice of the care of such patients on a global level [9]. Prospective studies from real-world AF cohorts from different geographic regions, especially in developing nations, could help explain the marked interregional variations in the clinical features, comorbid disease, and discrepancies in the incidence of long-term outcomes and establish practice options to reduce morbidity and mortality in patients with AF [8–11].

Middle Eastern studies of AF are scarce. The available studies were conducted before the widespread use of DOACs, and were limited by the small numbers of enrolled patients, and by the heterogeneous patient populations of native and workforce South Eastern Asian patients [12–17]. Furthermore, there is an existing limited data from the Middle East that address the long-term adherence to DOACs and its potential role to improve outcomes in patients with NVAF [17].

The Jordan AF (JoFib) study enrolled a Middle Eastern cohort of patients with valvular AF (VAF) and with NVAF during the contemporary era of increasing use of DOACs. The baseline features of the studied population, and the high rate of adherence to the use of OACs in general, and DAOCs in particular, according to recent practice guidelines were published earlier [18]. The current analysis presents the one-year outcome data from the JoFib study. We specifically report the use of, and adherence to OACs, and major events of all-cause and cardiovascular mortality, stroke and systemic embolization, bleeding events, and hospital admission.

## 2. Materials and Methods

The methods and baseline data from the JoFib have been described previously [18]. Briefly, consecutive adult patients (>18 years of age) with AF who were evaluated at 29 hospitals and out-patient cardiology clinics in Jordan were enrolled from May 2019 through October 2020. Diagnosis of AF was confirmed by 12-lead electrocardiogram (ECG), rhythm strip lasting >30 seconds, >1 episodes of AF on ambulatory ECG monitor, or a past diagnosis by a treating cardiologist. VAF was diagnosed in the presence of moderate to severe rheumatic mitral stenosis, or cardiac prosthetic mechanical valve. All other patients were classified as having NVAF. Patients were followed up prospectively for one year during outpatient clinic visits, hospital readmission, or phone calls at 1, 6, and 12 months after enrollment.

End points of interest were all-cause death, cardiovascular (CV) death, stroke, SE, major bleeding events, intracranial bleeding, nonmajor clinically significant bleeding events, and hospital admission for cardiac cause. CV deaths were defined according to standard definition, and included deaths that result from an acute myocardial infarction, sudden cardiac death, and death due to heart failure (HF), stroke, CV procedures, CV hemorrhage, and other CV causes [19]. Non-CV deaths were defined as deaths due to specific CV cause. Cardiac diseases that necessitated hospital admissions included acute coronary syndrome, recurrence of AF, ventricular tachyarrhythmias, symptomatic bradyarrhythmias, heart failure, and percutaneous or surgical coronary revascularization. Stroke was diagnosed based on neurologist evaluation based on standard clinical and imaging criteria. SE was diagnosed based on documented clinical, angiographic, intraoperative, or pathological evidence of atheroembolism to an arterial bed of the extremities or abdominal aorta branches. Stroke/SE risk was categorized according to the CHA2DS2-VASc score [20]. Low-risk score was defined as score 1 in women or 0 in men, moderate-risk score was defined as score 2 in women and 1 in men, and high-risk score was defined as score >3 in women and >2 in men [2]. Major bleeding event was defined based on the International Society of Thrombosis and Hemostasis definition, and included fatal bleeding, symptomatic bleeding in a critical area or organ (i.e., intracranial, intraspinal, intraocular, retroperitoneal, intraarticular, or pericardial), and/or bleeding that causes a fall in hemoglobin level of >2 g/dL or requires >2 units of whole blood or red cells transfusion [21]. Clinically relevant nonmajor bleeding was defined bleeding event that results in hospitalization, requires medical and/or surgical evaluation or intervention, or requires physician-directed change in antithrombotic regimen [21]. Institutional Review Board of participating centers approved the study. Patients signed a written informed consent. The study was registered at http://clinicatrials.gov (unique identifier number NCT03917992).

### 2.1. Statistical Analysis

Data were analyzed using IBM SPSS version 24. Descriptive statistics were performed using means and standard deviation (SD) to describe the continuous variables, and percentages were used to describe the categorical variables. Independent *t* test was used to compare means, and chi-square test was used to compare percentages. Multivariate binary logistic regression was conducted to determine factors associated with all-cause mortality and stroke/SE in patients with NVAF. The variables in the logistic regression model were selected using stepwise backward method. A *p* value of less than 0.05 was considered statistically significant.

## 3. Results and Discussion

### 3.1. Results

**Figure 1:**
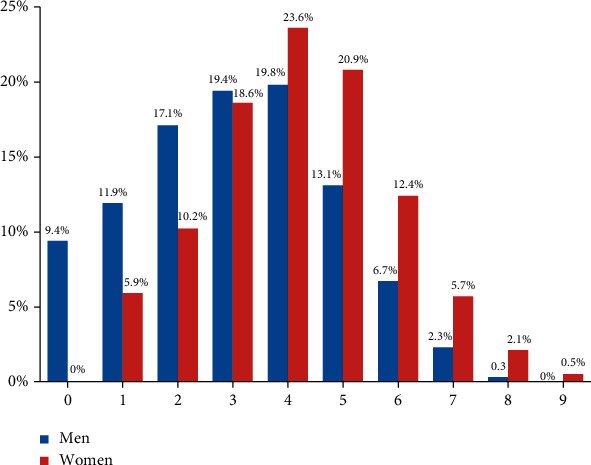
CHA2DS2-VASc score for men and women.

Of 2020 consecutive patients enrolled, 1849 (91.5%) patients had NVAF, and 171 (8.5%) patients had VAF. At one-year follow-up, a total of 116 (5.7%) patients were lost to follow-up, and 1904 (94.3%) patients had available data for analysis.  Table 1 shows the baseline demographic, clinical, and echocardiography features of the whole cohort and the NVAF and VAF subgroups. Compared with the VAF patients, NVAF patients were older, more likely to be men, and had higher prevalence of several cardiovascular risk factors, including hypertension, type 2 diabetes, and cigarette smoking. Furthermore, they were less likely to have nonparoxysmal AF and had higher mean HAB-BLED score compared with those with VAF. The mean (±SD) CHA2DS2-VASc score in NVAF patients was 4.1 ± 1.7 in women and 3.6 ± 1.8 in men. High-risk CHA2DS2-VASc score (score>3 in women and>2 in men) was present in 83.9% of women and 78.7% of men, intermediate-risk score (score 2 in women and 1 in men) was present in 10.2% women and 11.9% men, and low-risk score (score 1 in women and 0 in men) was present in 5.9% of women and 9.4% of men (Figure 1). The use of OACs, antiplatelet agents, and other medications in the whole cohort and in the two groups with NVAF and VAF is depicted in Table 2. Vitamin K antagonist (VKA) was prescribed for the majority of the patients with VAF, while OACs were prescribed for 78.6% of patients with NVAF (65.7% DOACs and 34.3% VKA). At one-year follow-up, 1725 (90.6%) patients were still using the same OAC agent, and 179 (9.4%) patients changed the prescribed OAC agent (59 patients switched VKA to DOAC, 23 patients switched DOAC to VKA or low-molecular weight heparin, 13 patients switched a DOAC agent to another, 57 patients temporary stopped taking OAC, and 27 patients discontinued OAC permanently). Antiplatelet therapy was used in 900 (44.6%) patients with NVAF, including one antiplatelet agent in 774 (38.3%) patients, and dual antiplatelet agents in 126 (6.2%) patients. Among patients with NVAF using OACs, concomitant single and dual antiplatelet agents were used in 28.8% and 3.3% of patients, respectively. Antiplatelet-only therapy was prescribed for 13% of patients with NVAF. Other medications used included antiarrhythmic agents in 21.1% of patients (class III (amiodarone) in 19.2% and class IC medications in 1.9% of patients), and rate control medications, with beta-blockers being the most commonly used in 80.1%.

The one-year event rates of major cardiovascular events are shown in Table 3. Overall, all-cause and CV mortality rates were 11.4% and 7.8%, respectively, and both of these events were significantly higher among patients with NVAF patients than VAF patients. Combined stroke (*N* = 75) and systemic embolization (*N* = 9) events occurred in 4.5% of patients. The majority of thromboembolic events (78.6%) occurred after the first month of follow-up. The incidence rate of major bleeding events was 2.6%, including 9 cases of intracranial hemorrhage (ICH). The majority of bleeding events (77.6%) also occurred after the first month of follow-up. In addition to ICH accounting for 19.1% of major bleeding events, other events included bleeding that led to hospital admission (40.4%), associated with hemoglobin drop (25.5%), and bleeding that required blood transfusion (14.9%). Nonmajor, clinically significant bleeding events were more common in patients with VAF than NVAF, and the most common minor bleeding events were skin bruises (38.7%), mucosal oral and nasal bleeds (37.2%), and hematuria (8.0%).

Of the whole cohort, 14.6% and 18.3% needed hospital admission for cardiac and noncardiac indications, respectively. The most common cardiac indications for hospital admission included heart failure (7.7%), dysrhythmias (4.1%), and acute coronary syndrome and coronary revascularization (2.8%). By the end of the study, 24 (1.3%) patients had electric cardioversion, 14 (0.7%) required permanent pacemaker, 6 (0.3%) patients needed AF ablation, and one (0.05%) patient had a left atrial occlusion device.

In the multivariate analyses of factors associated with all-cause death and stroke/SE in patients with NVAF (Table 4), the factors associated with all-cause death were in-patient enrollment setting (odds ratio [OR] 4.1), age>75 years of age (OR 2.5), and heart failure (OR 2.4). The use of OACs and body mass index <25 kg/m^2^ was significantly associated with lower odds of all-cause death. The strongest factors predictive of stroke/SE were high-risk CHA2DS2-VASc score (OR 5.8) and past history of stroke (OR 3.4).

### 3.2. Discussion

The Jordan AF study represents the first contemporary study that addresses management practices and major cardiovascular events after one-year of follow-up in a relatively large Middle Eastern cohort of patients enrolled in the era of an increasing utilization of the newer OACs to prevent stroke and SE. The principal findings of the study are as follows: (i) the majority of AF patients (9 of 10) had NVAF; (ii) the majority of patients with NVAF had high-risk CHA2DS2-VASc score (∼80%); and (iii) rates of incidence of major cardiovascular events at one-year of follow-up were comparable to those reported by studies from other regions in the world [22–24].

The AF population we studied was mostly patients with NVAF. This is a universal finding reported by other investigators and is explained by the global improved life expectancy and the increasing prevalence of CV risk factors that contribute to the pathogenesis of NVAF [4–6, 25]. Patients with NVAF in this study had high prevalence of CV risk factors and comorbidities; findings reflected by a mean CHA2DS2-VASc score are higher than that reported by the DOACs phase III clinical trials [26]. Despite reports of the notoriously suboptimal rates of utilization of, adherence to, and early discontinuation of OACs in NVAF patients [27, 28], we observed a reassuring high rate of utilization of OACs at enrollment and high rate of adherence to these medications at one year. Furthermore, the current study represents a significant disagreement with prior studies that addressed regional differences in the frequency of OACS use and showed highest uptake in Europe (90%) and lowest in Asia (57%) [29]. The high rate of utilization of DOACs compared to VKA in eligible patients with NVAF in the current study was mainly driven by high adherence rate to the most recent practice guideline at the time of the conduction of the study [2, 18].

Two small groups of NVAF patients in this study are worth focusing on. The first is the minority of OACs-eligible patients (10.0%) who were not prescribed these agents in the absence of a clinical contraindication to their use, and the second group is that of patients (3.0%) who received OACs despite having a low-risk CHA2DS2-VASc score. The reason behind these findings is not clear, but could be related to an exaggerated estimation of bleeding risk in the first group, and of thromboembolism in the second group.

Despite the current recommendations against the use of antiplatelet agents for the prevention of thromboembolism in patients with AF [30], these agents (commonly aspirin), rather than DOACs, were prescribed for 14% of patients with NVAF, a finding we share with other studies [31].

Our data confirm the considerable mortality and morbidity associated with AF, even in a contemporary clinical practice setting. The high one-year all-cause (11.4%) and cardiovascular (7.8%) mortality rates we reported are consistent with other studies from the Middle East, Asia, and other regions in the world, where all-cause mortality rates ranged between 5.2% and 15.3% and cardiovascular mortality rates ranged between 4.0% and to 7.0% [13–15, 32, 33]. The one-year risk of stroke/SE of 4.5% was not different from those reported by other studies [13, 23, 32]. The lower one-year rates of stroke/SE (<2%) reported by few investigators in the region were demonstrated in populations with lower CHA2DS2-VASc score [15] and by studies that involved single DOAC agent [14, 34]. The independent risk factors for all-cause mortality and for stroke/SE reported in patients with NVAF in this study were similar to other studies, namely, high-risk CHA2DS2-VASc score and past history of stroke which were the strongest two predictors of stroke/SE [32, 35].

The most feared bleeding event associated with the use of OACs in patients with AF is intracranial hemorrhage, which occurred in 9 (0.5%) patients at one year in the current study. This low rate and the low rate of major bleeding events (2.6%) are both reassuring and are in line with the results of other regional studies [26, 34, 36]. The burden of hospitalizations in patients with AF is a major source of an escalating health-care cost. Hospital admissions were common in our cohort at ∼15%., especially for heart failure and dysrhythmias.

Few limitations of this study worth of discussion are as follows: Studies with observational designs inherently have potential bias of residual confounding, data collection, and patient recall of events. This effect was overcome by recruitment of consecutive patients from different sectors of the local health-care system. The fact that the major cardiovascular events studied were hard endpoints, such as death, stroke, and major bleeding, that were very unlikely to be affected by recall issues. The number of patients lost to follow-up (5.7%) is lower than that reported by other investigators (>12% in European studies) [31, 34], but still may hamper the discriminatory ability of some analyses and the generalizability of the results. Despite these limitations, this study represents an important contribution to the contemporary knowledge of baseline features and one-year outcome in a Middle Eastern population with AF.

## 4. Conclusions

The Jordan AF study presents the first contemporary registry that focuses on baseline features, management practices, and one-year outcome in a Middle Eastern cohort with AF in the era of an increasing use of DOACs. Overall, one-year mortality and morbidity rates are comparable to those reported by other studies from the Middle East and other regions.

## Figures and Tables

**Table 1:** Baseline demographic, clinical, and echocardiography characteristics of 2020 patients with AF and two subgroups of NVAF and VAF.

Clinical feature	All AF cohort *N* = 2020	NVAF *N* = 1849 (91.5%)	VAF *N* = 171 (8.5%)	*P*∗
Age (years), mean + SD	76.9 + 13.0	68.4 + 12.9	61.0 + 12.0	<0.0001
Women, *N* (%)	1096 (54.3)	981 (53.1)	115 (67.3%)	<0.0001
Hypertension, *N* (%)	1506 (74.6)	1413 (76.2)	93 (54.4)	<0.0001
Type 2 diabetes mellitus, *N* (%)	882 (43.7)	826 (44.7)	56 (32.7)	0.03
Hypercholesterolemia, *N* (%)	280 (13.9)	253 (13.7)	27 (15.8)	0.52
Current cigarette smoking, *N* (%)	909 (45.0)	861 (46.6)	48 (28.1)	<0.0001
BMI (kg/m2), mean + SD	27.9 + 4.3	28.5 + 5.1	25.7 + 4.5	0.06
Prior or presenting stroke, *N* (%)	224 (11.1)	208 (11.2)	16 (9.4)	0.55
CHD, *N* (%)	215 (10.6)	205 (11.1)	10 (5.8)	0.04
Heart failure, *N* (%)	482 (23.9)	446 (24.1)	36 (21.1)	0.43
CKD, *N* (%)	181 (9.0)	172 (9.3)	9 (5.3)	0.11
Malignancy, *N* (%)	110 (5.4)	96 (5.2)	14 (8.2)	0.14
Nonparoxysmal AF	1292 (64.0)	1158 (62.6)	134 (78.4)	<0.0001
HAS-BLED score, mean + SD	1.7 + 1.1	1.7 + 1.1	1.3 + 1.1	<0.0001
Enrolled as outpatient setting, *N* (%)	1463 (72.4)	1312 (71.0)	151 (88.3)	<0.0001
LVEF <40%∗∗, %	17.8%	18.2%	14.4%	0.26
Left atrial diameter>3.5 cm∗∗, %	22.7%	16.1%	96.5%	<0.0001
LVH∗∗, %	39.0%	39.4%	35.1%	0.34
Pulmonary artery hypertension∗∗, %	25.3%	23.5%	45.3%	<0.0001

AF: atrial fibrillation; BMI: body mass index; CHD: coronary heart disease; CKD: chronic kidney disease; LVEF: left ventricular hypertrophy; LVH: left ventricular hypertrophy; NVAF: nonvalvular AF; VAF: valvular AF. ∗Difference between NVAF and VAF patients. ∗∗Transthoracic echocardiography was done for 90% of patients.

**Table 2:** Oral anticoagulant and antiplatelet agents and other cardiovascular medications in patients with AF and two subgroups of NVAF and VAF.

Medication	All AF cohort *N* = 2020	NVAF *N* = 1849 (91.5%)	VAF *N* = 171 (8.5%)	*P* value∗
Anticoagulant agents				
DOACs, *N* (%)	970 (48.0)	955 (51.6)	15 (8.8)	<0.0001
VKA, *N* (%)	649 (31.1)	499 (27.0)	150 (87.7)	<0.0001
LMWH, *N* (%)	46 (2.3)	46 (2.5)	0 (0)	0.07
None, *N* (%)	355 (17.6)	349 (18.9)	6 (3.5)	<0.0001
Antiplatelet agents				
Single agent, *N* (%)	774 (38.3)	726 (39.3)	48 (28.0)	0.01
Dual agents, *N* (%)	126 (6.2)	124 (6.7)	2 (1.2)	0.01
Single or dual agents, *N* (%)	900 (44.6)	850 (46.0)	50 (29.2)	<0.0001
Antiarrhythmic and rate control medications				
Amiodarone	387 (19.2)	358 (19.4)	29 (17.0)	0.51
Class I antiarrhythmic medications	38 (1.9)	36 (1.9)	2 (1.2)	0.72
Beta blockers, *N* (%)	1619 (80.1)	1474 (79.7)	145 (84.8)	0.13
Digitalis, *N* (%)	319 (15.8)	260 (14.1)	59 (34.4)	<0.0001
Nondihydropyridine CCB, *N* (%)	219 (10.8)	210 (11.4)	9 (5.3)	0.02
Other cardiovascular medications				
RAS inhibitors, *N* (%)	781 (38.7)	741 (40.1)	40 (23.4)	<0.0001
Diuretics, *N* (%)	792 (39.2)	712 (38.5)	80 (46.8)	0.04
Statins, *N* (%)	753 (37.3)	715 (38.7)	38 (22.2)	<0.0001

AF: atrial fibrillation; chronic kidney disease; CCB: calcium channel blocker; DOACs: direct oral anticoagulant agents, LMWH: low molecular weight heparin; LVH: left ventricular hypertrophy; NVAF: nonvalvular AF; VAF: valvular AF. RAAS: renin angiotensin system blockers; VKA: vitamin K antagonists. ∗Difference between NVAF and VAF patients. ∗∗Transthoracic echocardiography was done for 90% of patients.

**Table 3:** One-year outcome in the AF∗ cohort and the two groups with NVAF and VAF.

Event	All AF patients *N* = 1865	Patients with NVAF *N* = 1709	Patients with VAF *N* = 156	*P*
All-cause death, *N* (%)	218 (11.4)	210 (12.4)	8 (5.1)	0.01
Cardiovascular death, *N* (%)	146 (7.8)	127 (7.4)	3 (1.9)	0.02
Stroke/systemic embolism, *N* (%)	84 (4.5)	81 (4.7)	3 (1.9)	0.16
Major bleeding, *N* (%)	49 (2.6)	46 (2.7)	3 (1.9)	0.74
Intracranial bleeding, *N* (%)	9 (0.5)	9 (0.6)	0 (0)	0.69
Nonmajor clinically significant bleeding, *N* (%)	128 (6.9)	107 (6.3)	21 (13.5)	0.001
Hospital admission for cardiac cause, *N* (%)	272 (14.6)	247 (14.5)	25 (16.0)	0.70

AF: atrial fibrillation; NVAF: nonvalvular AF; VAF: valvular AF. ∗Calculations were performed after excluding the group that lost follow-up (*N* = 116) plus those who were enrolled as in-patients who died during the index hospitalization, and had no data at 1-, 6-, and 12-month follow-up (*N* = 39).

**Table 4:** Multivariate analysis of factors associated with all-cause death and stroke/SE among patients with nonvalvular atrial fibrillation.

Variable	Odds ratio	95% confidence interval	*P* value
All-cause death			
Type 2 diabetes	1.5	1.1-2.1	0.01
Age>75 years	2.4	1.6-3.5	<0.001
Enrolled as in-patient	4.1	3.0-5.6	<0.001
BMI<25 kg/m^2^	0.7	0.5-0.9	0.02
CHD	1.7	1.1-2.5	0.02
Heart failure	2.4	1.7-3.3	<0.0001
Use of OACs	0.4	0.3-0.6	<0.0001
Major bleeding	2.3	1.2-4.4	0.02
Stroke/SE			
Hypertension	1.9	1.0-3.8	0.05
Enrolled as in-patient	3.1	2.1-4.7	<0.001
CKD	1.8	1.1-3.1	0.02
High-risk CHA2DS2-VASc score	5.8	1.4-24.4	0.02
Prior history of stroke	3.4	2.3-5.2	<0.0001

BMI: body mass index; CHD: coronary heart disease; CKD: chronic kidney disease; OAC: oral anticoagulants; SE: systemic embolism.

## Data Availability

Readers can access the data supporting the conclusions of the study by contacting the corresponding author directly.
